# Prevalence and type distribution of human papillomavirus in a Chinese urban population between 2019 and 2023: a retrospective study

**DOI:** 10.3389/fmicb.2025.1735393

**Published:** 2026-01-09

**Authors:** Mei-Yan Xu, Mei-Jing Feng, Wen-Yao Geng, Xue-Kai Liu, Rui Ma

**Affiliations:** 1 Department of Nutrition, Aerospace Center Hospital, Beijing, China; 2 Department of Health Management, Aerospace Center Hospital, Beijing, China; 3 Department of Clinical Laboratory, Aerospace Center Hospital, Beijing, China

**Keywords:** China, COVID-19, human papillomavirus, prevalence, type distribution

## Abstract

**Introduction:**

Human papillomavirus (HPV) infection is one of the most common sexually transmitted infections among women worldwide. The present study reports the prevalence and type distribution of HPV in the same hospital between 2019 and 2023, and analyzes the impact of the COVID-19 pandemic on HPV infection.

**Methods:**

Female participants aged ≥18 years old were recruited from Aerospace Center Hospital in Beijing, China, between 2019 and 2023. A total of 27 HPV types were detected using fluorescence quantitative polymerase chain reaction.

**Results:**

37,225 women underwent at least one HPV test, with an overall positive rate of 10.22%. The HPV positive rates in the age groups of ≤45 years, 46–60 years, and >60 years were 9.44, 11.61, and 11.14%, respectively. By year, the positive rates were 5.19% in 2019, 11.60% between 2020 and 2022, and 9.37% in 2023. Women with uterine fibroids or a history of hysterectomy/ovariohysterectomy had higher HPV positive rates than those without these conditions. Compared with the pre- and post-COVID-19 periods, the HPV positive rate was significantly higher during the pandemic (2020–2022). Among high-risk HPV types, the top five most prevalent ones (by positive case count) were HPV52, 16, 58, 53, and 56. For low-risk HPV, HPV61 had the highest positive rate from 2020 to 2023. Single-type HPV infection accounted for the highest proportion across all age groups and years. Joinpoint regression analysis showed that the HPV positive rate decreased sharply in the 31–35 years age group (Annual Percent Change [APC] = −22.2%, 95% Confidence Interval [CI] = −27.9 ~ −13.1), then increased steadily until the 66–70 years age group (APC = 6.9%, 95% CI = 5.5 ~ 8.3), and declined again thereafter (APC = −18%, 95% CI = −25.4 ~ −7.3).

**Discussion:**

There may be a potential association between the COVID-19 pandemic and HPV transmission. Our findings provide valuable insights for optimizing HPV screening strategies and formulating HPV prevention measures. The association between gynecological diseases and specific HPV types requires further investigation.

## Introduction

Human papillomavirus (HPV) is the primary etiological agent of cervical cancer. Globally, there are over 570,000 new cases of cervical cancer and approximately 310,000 deaths annually, with 85% of these cases occurring in low-income countries (Sung et al., 2021). High-risk HPV (HR-HPV), particularly types 16 and 18, is responsible for 70% of cervical cancer cases, while HPV types 6 and 11 cause 90% of genital warts (de Sanjose et al., 2010). Due to inadequate screening coverage and low HPV vaccination rates, Africa and Asia bear the heaviest disease burden of HPV-related illnesses (World Health Organization Regional Office for Africa, 2024).

China is one of the regions with a high incidence of HPV-related diseases in Asia. It records approximately 110,000 new cervical cancer cases and 59,000 deaths each year, ranking second globally in both incidence and mortality (Cao et al., 2021). Currently, the estimated HPV infection rate in China ranges from 9.6 to 23.6% across different geographical regions. Women exhibit two age peaks for HPV infection: one in the group of ≤25 years and the other in the group of 50–60 years. Among the Chinese population, the most frequently detected HPV genotypes are HPV16, 52, and 58 (Zhao et al., 2024).

HPV vaccination is a key intervention for preventing HPV infection, anogenital warts, and HPV-related precancerous lesions and cancers. Currently, three types of HPV vaccines (bivalent, quadrivalent, and nonvalent) are approved for marketing worldwide. HPV vaccine coverage is significantly higher in middle- and high-income countries than in low- and middle-income countries, with only 15 countries achieving a 90% coverage rate for the first dose (Han et al., 2025). Following widespread vaccination, the infection rate of vaccine-targeted genotypes (e.g., HPV16/18) has decreased significantly, while the proportion of non-vaccine-targeted genotypes has increased-suggested a potential phenomenon of genotype replacement.

In China, the bivalent HPV vaccine was launched in 2016, followed by the nonvalent vaccine in 2018. Although a certain vaccination rate has been achieved among women, the impact of vaccination on the prevalent HPV genotypes in the female population remains to be fully elucidated. The COVID-19 pandemic may have affected HPV infection dynamics, screening services, and vaccination programs (Ronga et al., 2023; Zhang et al., 2024). A previous study showed that strict lockdown measures significantly reduced the HPV infection rate. During the pandemic, the most common HPV genotypes were HPV16, 52, 58, and 53; after the pandemic, the HPV infection rate rebounded rapidly but remained slightly lower than the pre-pandemic level (Liu et al., 2022). Additionally, HPV infection rates varied across different regions during and after the pandemic (Lin et al., 2024; Wang et al., 2024; Ke et al., 2025; Yu et al., 2025).

Our research team previously investigated the HPV infection status among female health check-up attendees at Aerospace Center Hospital in Beijing during 2014–2018. However, the changes in HPV infection patterns during and after the COVID-19 pandemic, as well as the combined effects of HPV vaccine rollout in China, have not been fully elucidated. We hypothesize that the HPV prevalence and genotype distribution in this Beijing-based cohort may have changed between 2019 and 2023 relative to the pre-pandemic period (2014–2018), with such changes potentially associated with the COVID-19 pandemic and the growing uptake of HPV vaccines. Given this research gap, the present study aimed to update the data on the HPV prevalence and genotype distribution among female health check-up attendees in Beijing, and to systematically assess the potential impacts of the COVID-19 pandemic and HPV vaccination on HPV infection dynamics. The findings of this study are expected to provide evidence-based support for optimizing HPV prevention and control strategies in the region.

## Materials and methods

### Study population

This was a hospital-based retrospective epidemiological study. Participants were recruited from Aerospace Center Hospital in Beijing, China, between January 2019 and December 2023. The study protocol was approved by the Ethics Committee of Aerospace Center Hospital (No.: 2025–023).

The inclusion criteria of participants were female gender, age ≥18 years old, and participation in annual health check-ups with at least one valid HPV test result. Pregnant and postpartum women were excluded.

### Sample collection and HPV detection method

Cervical exfoliated cell samples were collected by physicians during gynecological examinations. Most samples were tested on the same day of collection; if testing was delayed, samples were stored at −70 °C until analysis.

A total of 27 HPV types were detected using the HPV Nucleic Acid Genotyping Kit (Flow Cytometry Fluorescence Hybridization Method; Shanghai Toujing Biotechnology Co., Ltd., Shanghai, China). This kit detects 17 HR-HPV types (HPV16, 18, 26, 31, 33, 35, 39, 45, 51, 52, 53, 56, 58, 59, 66, 68, and 82) and 10 LR-HPV types (HPV6, 11, 40, 42, 43, 44, 55, 61, 81, and 83). The kit contains two types of probes and 28 classification microspheres (including one quality control microsphere). Probes targeting HPV DNA are coated on 27 classification microspheres.

The HPV detection process comprises the following steps. (1) Premix, primer mixture, and polymerase were mixed thoroughly and dispensed into PCR reaction tubes (15 μL per tube). (2) Samples (5 μL per tube) were added to the corresponding reaction tubes. After sealing, tubes were centrifuged at 2000 rpm for 10 s. (3) PCR amplification was performed in a PCR instrument. (4) Hybridization detection was conducted in the amplification product analysis area under the following conditions: denaturation at 95 °C for 5 min, hybridization at 48 °C for 30 min, and incubation at 48 °C for 15 min. (5) The hybridization plate was immediately transferred to a preheated multi-functional flow cytometric bead array instrument for detection and result reading. An HPV type was considered positive if the signal intensity of its specific probe exceeded 150. Quality control was implemented throughout the experiment, and all procedures were performed in accordance with the kit instructions.

### Statistical analysis

The positivity rate of HPV is calculated as the number of positive HPV test results divided by the total number of HPV tests performed. HPV prevalence and type distribution were described using positive rates/proportions and 95% confidence intervals (CIs). The chi-square test was used to compare HPV positive rates across different age groups (≤45 years, 46–60 years and >60 years) and years (2019, 2020–2022 and 2023). An HPV-positive sample refers to a sample in which the subject is infected with at least one type of HPV. A single infection is defined as an infection involving only one HPV type. A co-infection is defined as an infection with two or more HPV types (≥2 HPV types). Joinpoint Regression Program (Version 4.1.1.1) was used to model trends in HPV positive rates and calculate the annual percent change (APC) with 95% CIs. A positive APC indicated an increasing trend, while a negative APC indicated a decreasing trend. Statistical significance was set at *p* < 0.05 (two-tailed). All analyses were performed using Stata 17.0 software (Stata Corp LLC, College Station, TX, USA).

## Results

### Overall HPV prevalence

A total of 37,225 HPV tests were performed between 2019 and 2023. Of all the tests, 3,805 were HPV-positive, and the overall positive rate of HPV was 10.22%. The positive rates of HPV were 9.44% (2,153/22,803, 95% CI 9.07–9.83) in the group of ≤45 years, 11.61% (1,123/9,674, 95% CI 10.98–12.26) in the group of 46–60 years, and 11.14% (529/4,748, 95% CI 10.26–12.07) in the group of >60 years (Table 1). There was a significant difference in HPV positive rates across age groups (*p* < 0.001). The positive rates of HPV were 5.19% (220/4.243, 95% CI 4.54–5.90) in 2019, 11.60% (2,573/22,177, 95% CI 11.18–12.03) between 2020 and 2022, and 9.37% (1,012/10,805, 95% CI 8.82–9.93) in 2023 (Supplementary Table 1). A significant difference on the HPV positive rates was observed across years (*p* < 0.001).

**Table 1:** HPV detective rate of women in different groups.

Variable	Total	No. of positive	Rate (%)	95% CI	*p*
Age, years					<0.001
≤45	22,803	2,153	9.44	9.07–9.83	
46–60	9,674	1,123	11.61	10.98–12.26	
>60	4,748	529	11.14	10.26–12.07	
Year					<0.001
2019	4,243	220	5.19	4.54–5.9	
2020–2022	22,177	2,573	11.60	11.18–12.03	
2023	10,805	1,012	9.37	8.82–9.93	
Uterine fibroids					<0.001
Yes	33,445	3,354	10.03	9.71–10.36	
No	3,780	451	11.93	10.91–13.01	
Hysterectomy and ovariohysterectomy					0.003
Yes	34,883	3,523	10.10	9.79–10.42	
No	2,342	282	12.04	10.75–13.43	

The HPV positive rate was 11.93% (451/3,780, 95% CI 10.91–13.01) in women with uterine fibroids versus 10.03% (3,354/33,445, 95% CI 9.71–10.36) in those without (*p* < 0.001). For women with a history of hysterectomy/ovariohysterectomy, the positive rate was 12.04% (282/2,342, 95% CI 10.75–13.43), compared with 10.10% (3.523/34,883, 95% CI 9.79–10.42) in those without this history (*p* = 0.003).

### Distribution of HPV infection before, during and after the COVID-19 epidemic

**Figure 1:**
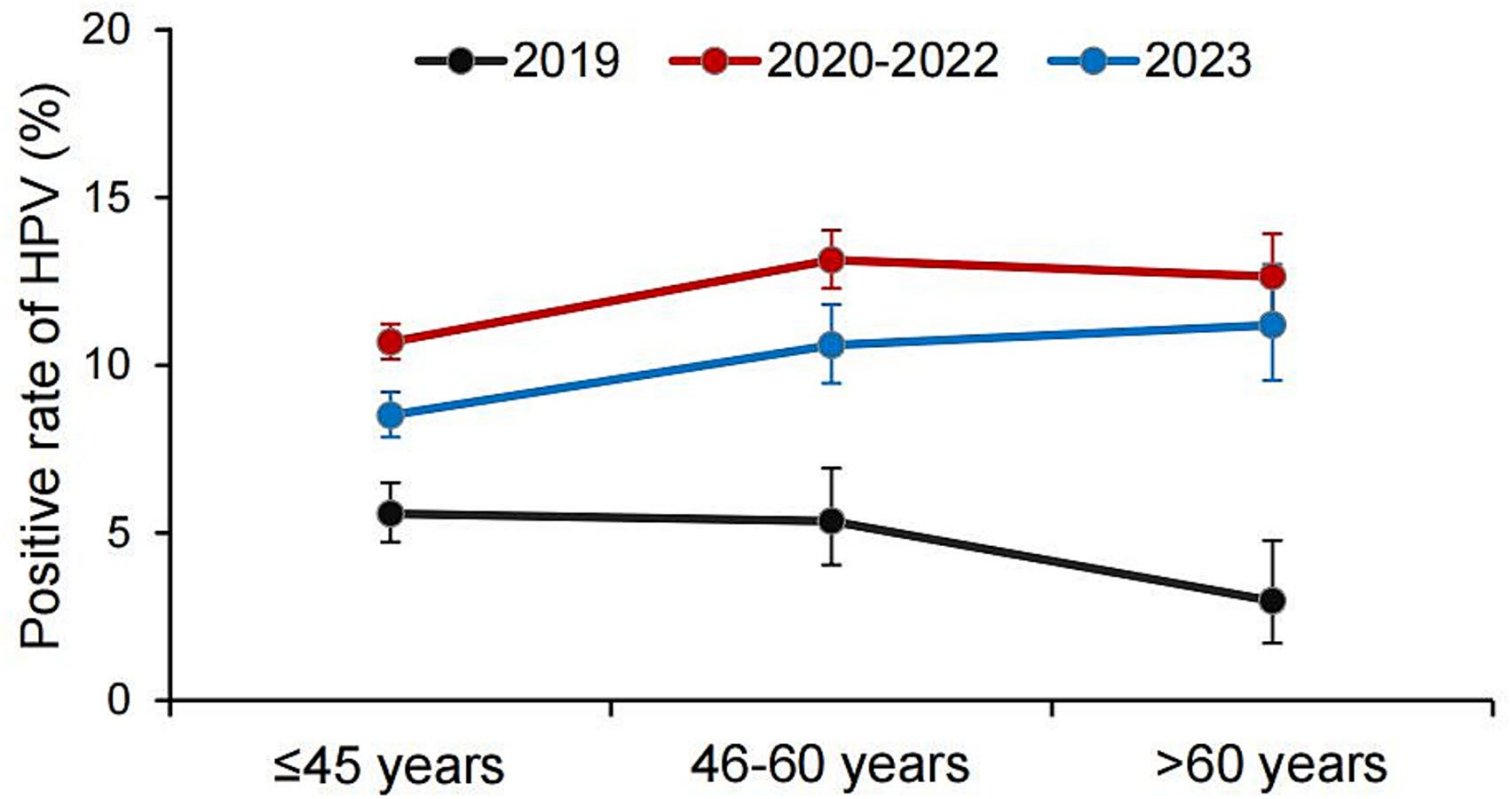
Positive rate of HPV before, during, and after the COVID-19 epidemic.

Before the COVID-19 pandemic (in 2019), the HPV positive rates among the age groups of ≤45 years, 46–60 years and >60 years were 5.57, 5.35, and 2.97%, respectively (Figure 1). During the COVID-19 pandemic (2020–2022), the corresponding HPV positive rates for these three age groups increased to 10.69, 13.14, and 12.65%, respectively. After the COVID-19 pandemic (in 2023), the HPV positive rates declined slightly to 8.51% (≤45 years), 10.59% (46–60 years), and 11.19% (>60 years) for the respective age groups.

### Distribution of HPV type infection

The overall positive rate of HR-HPV was 9.05% (3,368/37,225), while that of LR-HPV was 3.66% (1,363/37,225) (Supplementary Table 2). The highest prevalence of both HR-HPV and LR-HPV was observed during the 2020–2022 period, with this trend being particularly prominent among individuals aged ≤45 years.

**Figure 2:**
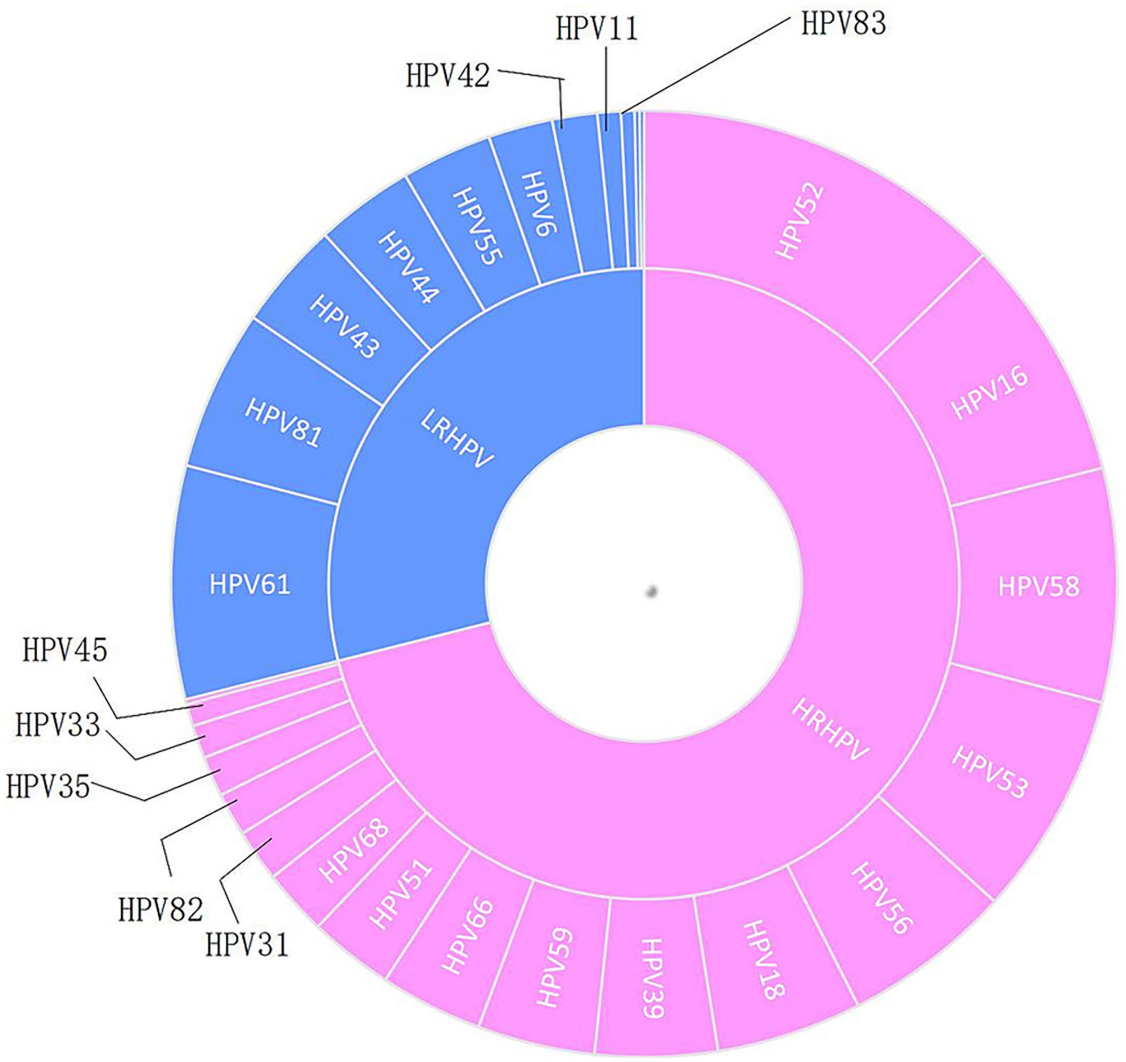
Positive number distribution of HRHPV and LRHPV. HRHPV, high-risk HPV; LRHPV, low-risk HPV.

In terms of HR-HPV, the top five types with the highest number of positive cases were HPV52, 16, 58, 53, and 56. For LR-HPV, the top five types with the highest positive rates were HPV61, 81, 43, 44, and 55 (Figure 2).

**Figure 3:**
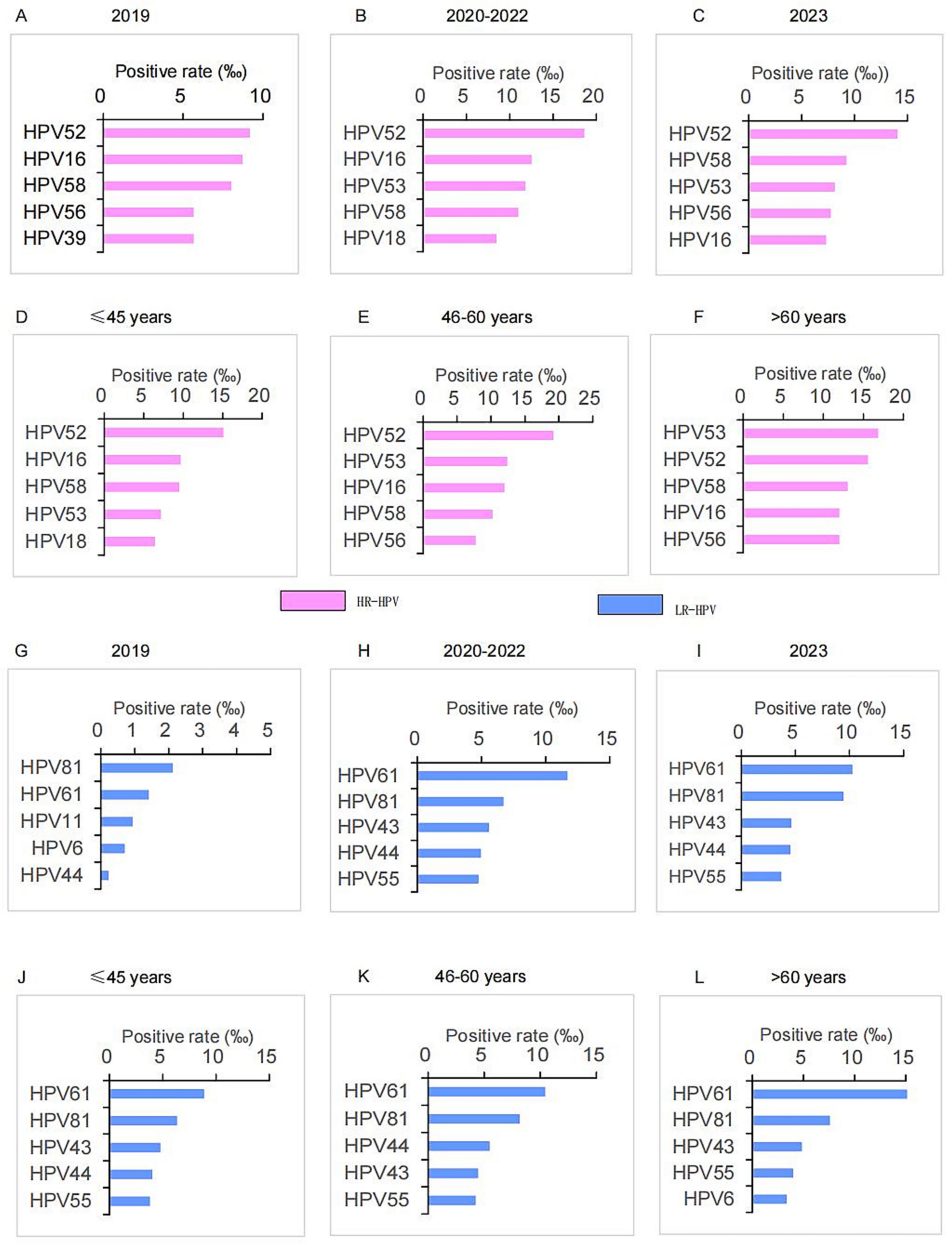
Top five types of HRHPV and LRHPV in different age and year groups. **(A–C)** Positive rate of top five HRHPV types in the year of 2019, 2020–2022 and 2023. **(D–F)** Positive rate of top five HRHPV types in the age of ≤45 years, 46–60 years, and >60 years. **(G–I)** Positive rate of top five LRHPV types in the year of 2019, 2020–2022 and 2023. **(J–L)** Positive rate of top five LRHPV types in the age of ≤45 years, 46–60 years and >60 years. HRHPV, high-risk HPV; LRHPV, low-risk HPV.

From 2019 to 2023, HPV52 had the highest positive rate among all HR-HPV types (Figures 3A–C). Specifically, HPV52 also ranked first in terms of positive rate in the groups of ≤45 years and 46–60 years (Figures 3D,E). In the group of >60 years, the top five HR-HPV types were HPV53, 52, 58, 16, and 56 (Figure 3F).

For LR-HPV, HPV81 had the highest positive rate in 2019, followed by HPV61, 11, 6, and 43 (Figure 3G). From 2020 to 2023, HPV61 became the LR-HPV type with the highest positive rate (Figures 3H,I). Notably, HPV61 maintained the highest positive rate among LR-HPV types across all age groups (Figures 3J–L).

### Single infection and co-infection of HPV

Among all 3,805 HPV-positive women, 3,100 had a single HPV infection, and 705 had a co-infection (Supplementary Table 3). By year, the number of single infection cases was 159 in 2019, 2,123 between 2020 and 2022, and 818 in 2023; in contrast, the number of co-infection cases was 61 in 2019, 450 between 2020 and 2022, and 194 in 2023, respectively.

**Figure 4:**
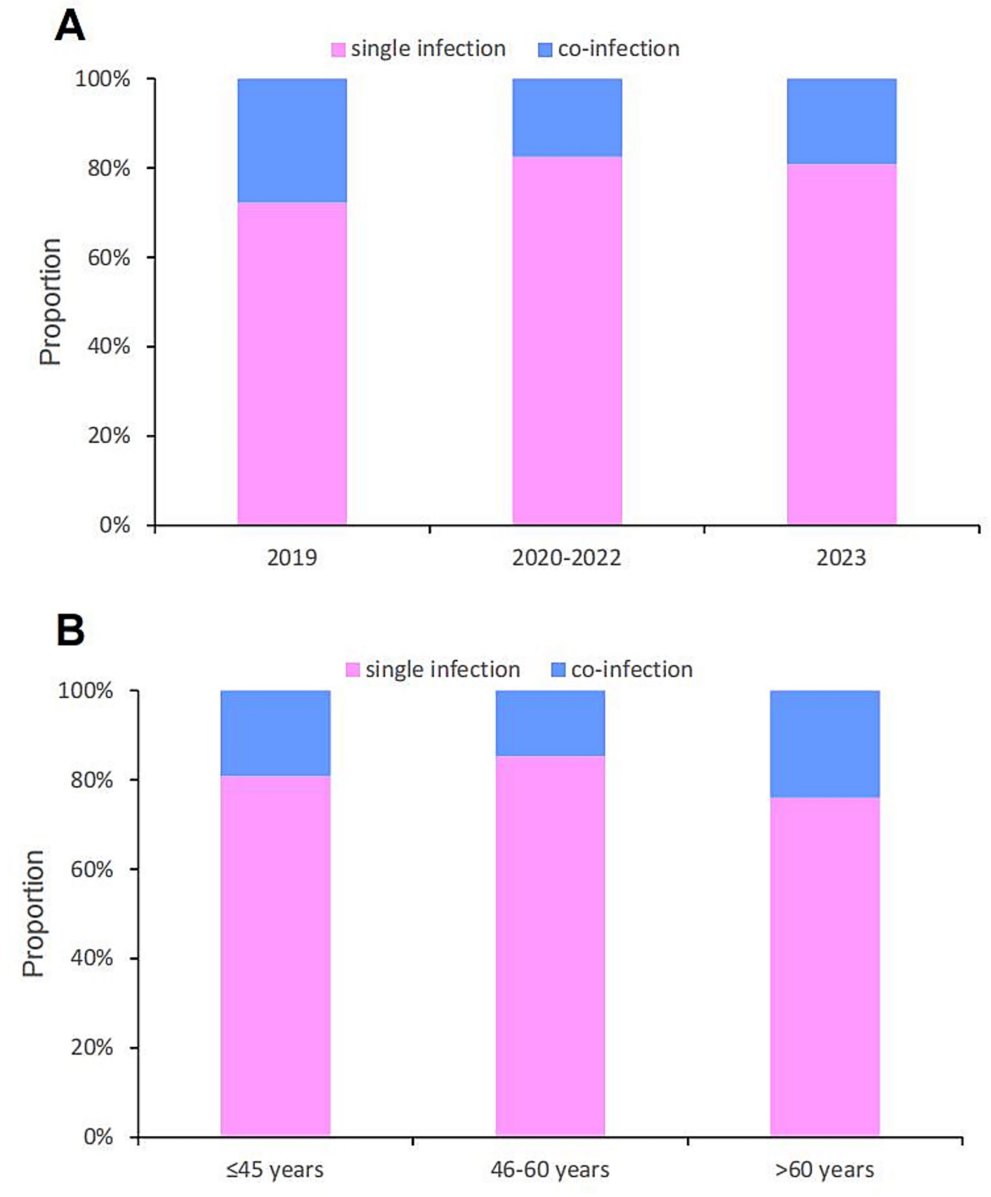
Single infection and co-infection proportion of HPV in different groups: **(A)** year groups; **(B)** age groups.

The number of single infection cases was 1,740 in the group of ≤45 years, 958 in the group of 46–60 years, and 402 in the group of >60 years; meanwhile, the number of co-infection cases was 413, 165, and 127 in these three age groups, respectively. Notably, single HPV infection accounted for the highest proportion across all age groups (Figure 4A) and year groups (Figure 4B).

### Dynamic changes of HPV positive rate

**Figure 5:**
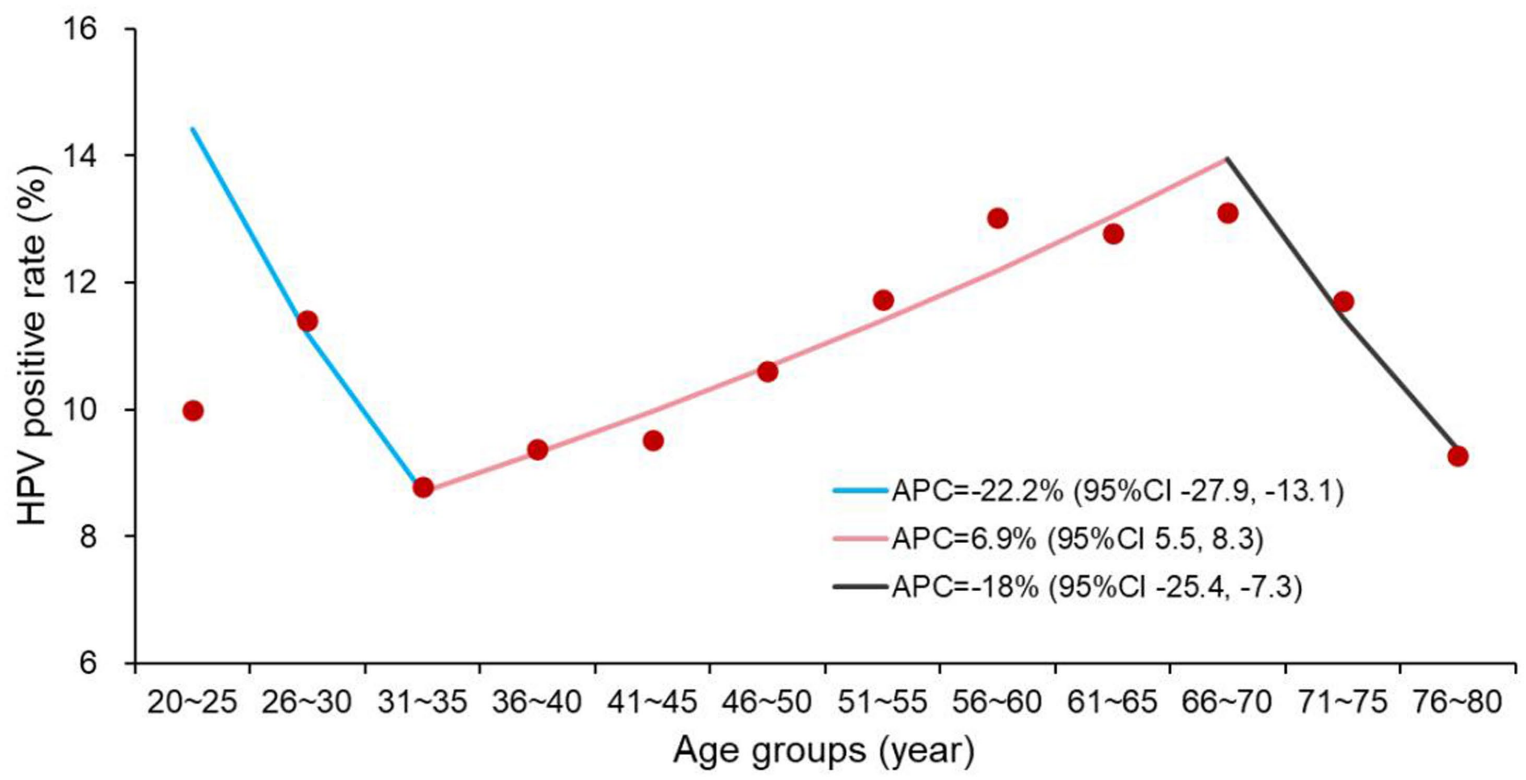
The trend of HPV positive rate along with the increase of age.

The HPV positive rate declined sharply in the group of 31–35 years (APC = −22.2%, 95% CI = −27.9 ~ −13.1), then increased steadily until the 66–70 years age group (APC = 6.9%, 95% CI = 5.5 ~ 8.3). After that, the HPV positive rate decreased again (APC = −18%, 95% CI = −25.4 ~ −7.3) (Figure 5).

## Discussion

For women undergoing health examinations in Beijing, this study revealed an overall HPV positive rate of 10.22% between 2019 and 2023. In comparison, a separate study reported an HPV positive rate of 17.5% in Sichuan Province during the same period (Mo et al., 2024), while another study showed a rate of 13.53% in the plateau region of Southwest China from 2014 to 2023 (Hu et al., 2024). However, among women attending routine outpatient gynecological examinations, the overall HPV positive rate was substantially higher which reached 26.92% in Jiangsu Province from 2013 to 2017 (Zhang et al., 2019), 22.51% in Chengdu from 2015 to 2020 (Wang et al., 2024), and 23.8% in Hangzhou from 2017 to 2023 (Ke et al., 2025). These findings indicate that HPV positive rates vary across different regions and time periods, as well as between populations (health examination attendees vs. gynecological outpatients).

Our research team previously conducted a similar study among female health examination attendees at the same hospital (Aerospace Center Hospital) between 2014 and 2018, which revealed that HPV positive rates remained stable at no more than 8.85% over this five-year period (Xu et al., 2020). In contrast, the present study showed notable fluctuations in HPV positive rates during the 2019–2023 period: after 2019, the rate rose significantly to 11.6% between 2020 and 2022 (a period coinciding with the COVID-19 pandemic) and then declined to 9.37% in 2023 (post-pandemic). However, some studies have reported the opposite trend: a decrease in HPV infection rates during the pandemic among outpatient and inpatient populations (Zhang et al., 2024; Yi et al., 2023). These conflicting findings suggest that the impact of the COVID-19 pandemic on HPV positive rates varies across different populations.

HPV positive rates also exhibited variations across different age groups. In the present study, the HPV positive rate declined sharply from the group of <20 years to the group of 31–35 years, then increased steadily until reaching the group of 66–70 years, and subsequently declined again. This age-related trend aligns with findings from our previous study conducted at the same hospital, which showed that individuals aged 20–29 years and 50–59 years were more susceptible to HPV18 infection. The consistency between the two studies further supports the presence of this distinct age distribution pattern in HPV positivity.

Other studies have reported varying patterns of age-specific HPV prevalence. One study identified the peak of HPV infection in individuals younger than 20 years old (Hu et al., 2024), while a study focusing on women in Ningbo Province, China, concluded that the peak HPV prevalence (17.9%) was observed in the group of 40–49 years (Hong et al., 2015). Another study showed a U-shaped curve for age-specific HR-HPV prevalence, with higher rates in the groups of ≤19 years old and ≥50 years old compared to other age brackets (Wang et al., 2019).

Additionally, a study conducted in Yunnan, southwestern China, reported that the highest HPV prevalence was seen in women aged ≤29 years old in urban areas and ≥50 years old in rural areas (Balocha et al., 2017). These findings highlight inconsistencies in the age distribution of HPV across different studies. Beyond age, several factors may contribute to the variations in HPV prevalence and distribution, including geographical location (e.g., rural vs. urban areas) and availability of healthcare resources. Other potential risk factors include marital status, sexual activity patterns, genetic variants, and co-infections with other diseases (e.g., HIV). Meanwhile, educational level, knowledge of public health, alcohol consumption, and tobacco smoking may act as confounding factors in these analyses (Hong et al., 2015; Balocha et al., 2017).

In the present study, the total positive rates of HR-HPV and LR-HPV were 9.05 and 3.66%, respectively, during the 2019–2023 period. Among LR-HPV types, HPV61 was nearly the predominant type across all age groups. For HR-HPV, HPV52 remained the most prevalent type--consistent with the findings of our previous study conducted at the same hospital from 2014 to 2018 (Xu et al., 2020), and aligned with results from other relevant research (Hu et al., 2024; Zhang et al., 2024), However, some studies have reported differing prevalence patterns: for example, HPV16 and HPV42 were observed to be more prevalent than HPV52 in their respective study populations (Yi et al., 2023; Rogua et al., 2023; Del Prete et al., 2017). This discrepancy in dominant HPV types may reflect variations in regional demographics, screening practices, or population-specific risk factors.

Globally, there are remarkable regional differences in the distribution of HPV types, which may be associated with a variety of factors, including local sanitation conditions, public awareness of sexual health, and the immune status of the population. In many developing countries in Africa and Asia, the infection rates of HR-HPV are relatively high. For instance, in specific regions of India, the infection rates of HPV16 and HPV18 can reach as high as 20–30% (Singh et al., 2015). In contrast, HPV16 and HPV18 remain the predominant types in developed countries such as those in Europe and the United States, and the overall HPV infection rate has declined in these regions largely due to the widespread promotion and uptake of HPV vaccination (Ährlund-Richter et al., 2019).

Within China, the distribution of HPV types also varies across different regions. In large cities, for example, the HPV infection rate tends to be relatively lower, which may be attributed to the higher health awareness among the local population (Xu et al., 2020). However, in certain economically developed regions, the detection rates of highly prevalent HPV types (such as HPV16 and HPV58) remain comparatively high. This phenomenon may be linked to large-scale population mobility and a relatively open attitude toward sexual life in these areas (Zhang et al., 2019). In contrast, in some economically underdeveloped inland regions, the incidence of cervical lesions caused by HR-HPV infections is relatively high. This is likely due to limited access to medical resources and poor hygienic conditions in these areas (Hu et al., 2024).

China is a multi-ethnic nation, and disparities also exist in the distribution of HPV types across different ethnic groups. For example, in regions with large ethnic minority populations, the distribution of HPV types exhibits unique characteristics, which are influenced by traditional lifestyles and customs. Among women of certain ethnic minorities-most notably Uyghur women-the infection rate of HPV31 tends to be higher (Pan et al., 2017), Potential factors contributing to this trend include the marital customs and fertility-related beliefs specific to this ethnic group. Nevertheless, with the increasing frequency of exchanges between different ethnic groups and the widespread dissemination of health-related knowledge, these ethnic disparities in HPV type distribution are gradually diminishing.

The present study showed that single HPV infections accounted for the highest proportion across all age groups and year groups. This finding is consistent with results from other regional studies in China. For instance, a study conducted in the plateau region of southwestern China reported that among HPV-positive cases, single infections accounted for 79.90%, followed by double infections (15.17%), triple infections (3.59%), and quadruple or more infections (1.33%) (Hu et al., 2024). Another study, which focused on Chengdu and Aba District in Sichuan Province, China, also reported that single infections (alongside HR-HPV infections) were the most common types of HPV infection (Wang et al., 2024). Collectively, almost all of these studies have reached a consistent conclusion: single HPV infections are the most prevalent form of HPV infection across different populations and regions.

Squamous precancerous and carcinomatous lesions of the female anogenital tract and cervix are predominantly caused by transforming infections with HR-HPV. Among HR-HPV types, HPV16 and HPV18 are the most prevalent oncogenic genotypes, accounting for approximately 70% of cervical cancer cases worldwide (Ogembo et al., 2015). Additionally, certain cervical adenocarcinomas are associated with persistent infections of specific HR-HPV types-including HPV16, 18, 31, 33, 51, and 52-which are closely linked to the development of cervical cancer (Schmidt, 2013). The present study showed that individuals diagnosed with uterine fibroids had a higher HPV positive rate. However, there is no direct causal relationship between uterine fibroids and HPV infection that uterine fibroids are hormone-dependent tumors, whereas HPV is primarily associated with epithelial cell lesions in the cervix, vagina, and other related sites. Additionally, the observed association may be linked to healthcare-seeking behavior. Specifically, patients with uterine fibroids may undergo more frequent or comprehensive gynecological examinations (including HPV screening) due to their underlying condition, which could increase the likelihood of detecting HPV infections compared to the general population. In clinical research, when conducting comprehensive examinations for gynecological diseases, uterine fibroids and HPV infection may be detected concurrently. Notably, in some studies focused on cervical cancer, cervical tissues from patients with uterine fibroids are often selected as control samples to investigate HPV status across different tissue types (Zhai et al., 2017; Cui et al., 2022).

Hysterectomy with adnexectomy is primarily used to treat severe uterine or ovarian diseases, such as endometrial cancer and uterine sarcoma. If a patient is concurrently infected with HPV-particularly when persistent infection with HR-HPV types has led to cervical precancerous lesions or cervical cancer-it may be necessary to remove the uterus and other relevant tissues. However, for simple HPV infections that have not yet caused high-grade cervical lesions or cancer, hysterectomy with adnexectomy may not be required. Nevertheless, our research demonstrated that the HPV positive rate is significantly higher in the population who have undergone hysterectomy with adnexectomy. The exact reasons for undergoing hysterectomy with adnexectomy in this population require further investigation.

This study has several limitations. First, it was a single-center study limited to health check-up attendees at one hospital, which may restrict the generalizability of the results to all females in Beijing. Due to the impact of the COVID-19 pandemic, many individuals may have canceled or postponed their health check-ups, leading to a reduction in the number of participants who underwent physical examinations in 2020 and thus contributing to the instability of the results. Second, the lack of data on HPV vaccination status is critical considering China’s rollout of bivalent HPV vaccine in 2016 and nonavalent HPV vaccine in 2018. Vaccination serves as a major confounder for HPV genotype distribution and may hinder the interpretation of epidemiological trends. Additionally, the study lacks data on sexual behavior, other sexually transmitted infections, socioeconomic status, and relevant clinical information such as cytology results. This lack of data limits our ability to conduct in-depth analyses of the risk factors associated with HPV infection. Third, this study’s cohort of female annual health check-up attendees, as a specific subpopulation with greater health awareness and potential socioeconomic differences from the general female population or gynecological outpatients, introduces selection bias that limits the findings’ generalizability. Fourth, potential bias may be introduced in the handling of repeated measurement data from the same subjects, which could affect the reliability of the results.

There may be a potential association between the COVID-19 pandemic and HPV transmission. Our findings provide valuable insights for optimizing HPV screening strategies and formulating HPV prevention measures. The association between gynecological diseases and specific HPV types requires further investigation.

## Data Availability

The original contributions presented in the study are included in the article/Supplementary material, further inquiries can be directed to the corresponding authors.
