# Supplementary material for: Prevalence and type distribution of human papillomavirus in a Chinese urban population between 2019 and 2023: a retrospective study
Source: Front Microbiol. 2026 Jan 9;16:1735393. doi: 10.3389/fmicb.2025.1735393 (PMC12827778; doi:10.3389/fmicb.2025.1735393)
Supplement: Supplementary file 1 [file Data_Sheet_1.doc]

**Supplement Table 1. HPV positive rate in different years**

| Year | Total | No. of positive | Rate (%) | 95% CI | P |
| --- | --- | --- | --- | --- | --- |
| 2019 | 4243 | 220 | 5.19 | 4.53-5.90 | <0.001 |
| 2020 | 3688 | 520 | 14.10 | 12.99-15.26 |
| 2021 | 6494 | 696 | 10.72 | 9.98-11.50 |
| 2022 | 11995 | 1357 | 11.31 | 10.75-11.89 |
| 2023 | 10805 | 1012 | 9.37 | 8.82-9.93 |
