# Supplementary material for: Prevalence and type distribution of human papillomavirus in a Chinese urban population between 2019 and 2023: a retrospective study
Source: Front Microbiol. 2026 Jan 9;16:1735393. doi: 10.3389/fmicb.2025.1735393 (PMC12827778; doi:10.3389/fmicb.2025.1735393)
Supplement: Supplementary file 2 [file Data_Sheet_2.doc]

**supplemental table 2. Positive number of HPV in different HPV types**

| **HPV type** | **total** | **2019** | **2020-2022** | **2023** | **≤45** | **45-60** | **>60** |
| --- | --- | --- | --- | --- | --- | --- | --- |
| **HRHPV** |  |  |  |  |  |  |  |
| HPV52 | 604 | 34 | 278 | 79 | 221 | 116 | 57 |
| HPV16 | 394 | 14 | 188 | 36 | 146 | 72 | 18 |
| HPV58 | 378 | 14 | 5 | 2 | 6 | 2 | 0 |
| HPV53 | 364 | 9 | 65 | 13 | 52 | 27 | 6 |
| HPV56 | 275 | 1 | 33 | 12 | 27 | 17 | 10 |
| HPV18 | 236 | 1 | 40 | 16 | 37 | 17 | 11 |
| HPV39 | 198 | 1 | 125 | 49 | 120 | 54 | 24 |
| HPV59 | 190 | 37 | 18 | 9 | 22 | 10 | 4 |
| HPV66 | 167 | 39 | 83 | 40 | 95 | 25 | 17 |
| HPV51 | 137 | 10 | 413 | 152 | 344 | 186 | 74 |
| HPV68 | 107 | 24 | 262 | 88 | 164 | 120 | 80 |
| HPV31 | 85 | 16 | 167 | 84 | 143 | 75 | 57 |
| HPV82 | 70 | 16 | 244 | 100 | 217 | 99 | 62 |
| HPV35 | 65 | 24 | 119 | 55 | 122 | 34 | 34 |
| HPV33 | 54 | 6 | 110 | 41 | 87 | 54 | 26 |
| HPV45 | 36 | 9 | 71 | 26 | 71 | 21 | 15 |
| HPV26 | 8 | 12 | 44 | 20 | 48 | 16 | 6 |
| Total HRHPV | 3368 | 267 | 2265 | 822 | 1922 | 945 | 501 |
| **LRHPV** |  |  |  |  |  |  |  |
| HPV61 | 377 | 6 | 260 | 111 | 203 | 101 | 73 |
| HPV81 | 260 | 9 | 149 | 102 | 145 | 79 | 36 |
| HPV43 | 175 | 1 | 124 | 50 | 109 | 43 | 23 |
| HPV55 | 147 | 1 | 106 | 40 | 87 | 41 | 19 |
| HPV6 | 104 | 3 | 63 | 38 | 62 | 26 | 16 |
| HPV44 | 160 | 1 | 110 | 49 | 92 | 53 | 15 |
| HPV42 | 73 | 0 | 51 | 22 | 37 | 1 | 6 |
| HPV83 | 22 | 0 | 17 | 5 | 10 | 8 | 4 |
| HPV11 | 38 | 4 | 18 | 16 | 26 | 9 | 3 |
| HPV40 | 7 | 0 | 4 | 3 | 5 | 1 | 1 |
| Total LRHPV | 1363 | 25 | 902 | 436 | 776 | 362 | 196 |
