# Supplementary material for: Prevalence and type distribution of human papillomavirus in a Chinese urban population between 2019 and 2023: a retrospective study
Source: Front Microbiol. 2026 Jan 9;16:1735393. doi: 10.3389/fmicb.2025.1735393 (PMC12827778; doi:10.3389/fmicb.2025.1735393)
Supplement: Supplementary file 3 [file Data_Sheet_3.docx]

| **Variable** | **No. of single infection** | **No. of co-infection** | **Total** |
| --- | --- | --- | --- |
| **Age, years** |  |  |  |
| 2019 | 159 | 61 | 220 |
| 2020-2022 | 2123 | 450 | 2573 |
| 2023 | 818 | 194 | 1012 |
| **Year** |  |  |  |
| ≤45 years | 1740 | 413 | 2153 |
| 46-60 years | 958 | 165 | 1123 |
| >60 years | 402 | 127 | 529 |
| **Total** | 3100 | 705 | 3805 |

**Supplemental table 3. The number of single infection and co-infection of HPV in different age and year groups**
